# Host Filtering Shapes the Soil–gut Microbiome Linkages in Pastoral Systems

**DOI:** 10.1007/s00248-026-02791-6

**Published:** 2026-05-21

**Authors:** Upulika Jayaneththi, Nicholas W. Sneddon, Lucy L. Burkitt, Paramsothy Jeyakumar, Christopher W. N. Anderson, Lisanne M. Fermin, Daniel J. Donaghy

**Affiliations:** 1 School of Agriculture and Environment, Massey University, Palmerston North, New Zealand; 2 School of Veterinary Science, Massey University, Palmerston North, New Zealand; 3 Department of Agricultural Engineering and Soil Science, Faculty of Agriculture, Rajarata University of Sri Lanka, Puliyankulama, Anuradhapura, Sri Lanka

**Keywords:** Ruminant gut microbiome, Shared bacterial taxa, Soil-gut interactions, Soil microbiome

## Abstract

Soil and ruminant gut microbiomes are linked within grazing pastoral systems, yet the strength and direction of these connections under different pasture management systems remain poorly understood. This study characterised bacterial communities in soil and faeces from cattle and sheep grazing standard and diverse pastures managed under contemporary and regenerative practices using 16 S rRNA gene sequencing. Shared bacterial taxa between soil and gut were identified, and their dominance patterns and soil–gut diversity relationships were evaluated using Spearman correlations and Procrustes analyses. Several taxa, including *Prevotella copri*, *Faecalibacterium prausnitzii*, *Akkermansia muciniphila*, *Bacteroides uniformis*, *Clostridium perfringens* and *Fibrobacter succinogenes* were commonly shared between soil and gut but were usually more prevalent in the gut, indicating predominant host‑associated dominance. Soil exhibited significantly greater bacterial alpha diversity than the gut across all treatments, and neither pasture diversity nor management significantly affected soil–gut alpha diversity differences or the contributions of shared taxa. Correlations between soil and gut alpha diversity were weak and non-significant, and correlations for beta diversity between soil and gut varied through time and among treatments without reaching significance. These findings indicate that, although soil–gut microbial connectivity exists, gut communities are primarily shaped by host-related ecological processes and internal filtering, with soil acting mainly as a diverse reservoir and sink for gut-derived taxa. This highlights that host-driven gut microbiomes primarily shape the soil–gut microbiome link, limiting the strong direct influence of pasture diversity or management.

**Supplementary Information:**

The online version contains supplementary material available at 10.1007/s00248-026-02791-6.

## Introduction

Pastoral grazing systems are complex agroecosystems in which soil, plants, animals, and microorganisms are tightly interconnected through biological, chemical, and physical processes [1]. These interactions regulate essential soil and animal functions and underpin the long-term sustainability of pasture-based grazing systems [2, 3].

A key pathway within this interconnected system is the continuous transfer of microorganisms along the soil–plant–animal continuum [4]. Soil microbes influence plant nutrition and health, and plants mediate the quality and composition of the herbage consumed by grazing animals [5]. Microorganisms from soil and plant surfaces can therefore enter the gastrointestinal tract of ruminants, where they interact with and potentially shape the gut microbiome [4, 5].

The ruminant gut microbiome is fundamental to host nutrition and productivity, facilitating the degradation of fibrous plant material, synthesis of volatile fatty acids, and modulation of host immunity [6]. Conversely, grazing ruminants contribute to soil microbial dynamics through faecal deposition, returning both nutrients and microbial taxa to the soil and creating reciprocal feedback between the two microbiomes [4]. Key functional taxa such as mucin-degraders (*Akkermansia muciniphila*), cellulose-utilisers (*Fibrobacter succinogenes*), plant polysaccharide fermenters (*Prevotella copri*), and soil return taxa (*Clostridium perfringens*) can mediate these transfers [4, 7–9]. This bidirectional linkage has implications for both soil health and animal performance in pastoral systems [5].

The strength of soil–gut microbial connectivity is shaped by environmental conditions and management practices [5]. Soil microbial communities are influenced by pH, moisture, temperature, nutrient availability, grazing intensity, and plant composition. Among these drivers, pasture diversity and management practices are particularly important [4]. Pasture diversity influences root exudation, litter quality, and plant secondary metabolites, which in turn shape soil microbial composition, diversity, and function. Increased soil microbial diversity can enhance nutrient cycling efficiency, suppress pathogens, and improve plant resilience to stress [5, 10]. These changes may improve forage quality and potentially support a more functionally diverse ruminant gut microbiome [5]. Regenerative practices characterised by hyper-diverse pasture swards, longer rest periods between rotational grazing, longer pasture residual post grazing and minimal use of mineral fertiliser and chemical inputs, may further promote these soil-gut microbial interactions compared with intensive, plants-limited contemporary systems [11].

However, despite this apparent connectivity, soil and gut environments differ markedly in oxygen levels, pH, and nutrient availability, leading to distinct microbial communities [12, 13]. Most soil microorganisms are aerobic and cannot survive or remain metabolically active for extended periods in the anaerobic gut [14]. Under typical pastoral conditions the potential contribution of ingested soil microbes to the ruminant gut has been estimated at approximately 8% in sheep and 2.6% in cattle [15]. These estimates align with a global rumen survey reporting that only a small proportion (approximately 3%) of the rumen microbiome originates from microbes ingested via plants, soil, or water [16]. Although these proportions are relatively small, they may still have functional importance, particularly in diverse pasture systems where soil microbial diversity is elevated. In such systems, transferred beneficial taxa may enhance gut functions and animal productivity compared with pastures of limited plant diversity.

Few studies have explored soil-gut microbial interactions, and these are mostly based on controlled feeding experiments [17, 18]. Xin et al. (2022) [17] demonstrated that supplementing Tibetan sheep diets with up to 20% soil (optimal at 10%) significantly altered rumen bacterial communities, enhanced nutrient digestibility, and improved growth performance. Similarly, Wang et al. (2023) [18] showed soil intake during grazing modified rumen bacterial abundance and fermentation parameters in sheep, highlighting soil microbial role in rumen function. These studies provide initial evidence that soil microbes can influence gut microbial shifts and animal nutrition.

However, no studies conducted under pastoral systems have characterised the specific interacting microbial species, their dominance patterns, or diversity dynamics within soil-gut pathways during grazing. This represents a critical knowledge gap and limits our understanding of how management-driven shifts in soil microbial communities translate into changes in gut functions and animal productivity. Addressing this gap is essential for developing management strategies that optimise both soil health and ruminant performance.

This study addresses these gaps by characterising shared microbial taxa, dominance patterns, and diversity relationships between soil and gut microbiomes across contrasting pasture management practices in New Zealand. We compared diverse and standard pastures (ryegrass-clover dominant) under regenerative and intensive contemporary management practices in both cattle and sheep. We hypothesised that regenerative management with diverse pastures strengthens soil–gut microbial connectivity through enhanced transfer of beneficial taxa, leading to improved soil functionality, gut fermentation efficiency, animal productivity, and overall system resilience compared with contemporary systems.

## Methods

### Experimental Sites and Treatments

The experiments were conducted at Massey University Dairy Farm 1 (40° 22′ 35.5″ S, 175° 37′ 15.7″ E) and the Pasture and Crop Research Unit (PCRU; 40° 23′ 20.1″ S, 175° 36′ 40.8″ E) in Palmerston North, New Zealand. The region has a temperate climate. Long-term (30 years) climate records range from approximately 20–22 °C in summer to around 12 °C in winter, with a mean annual rainfall of 984 mm [19].

The study was conducted as part of the Whenua Haumanu research programme, a comprehensive seven‑year programme initiated in April 2022 that compares diverse and standard pastures under regenerative and contemporary management in dairy and sheep systems [20]. The third experimental year of the Whenua Haumanu programme was selected for this study, covering June 2024 – May 2025 for dairy systems and January – December 2024 for sheep systems.

At Dairy Farm 1, three separate dairy cow herds (Holstein–Friesian, Jersey, and Holstein–Friesian × Jersey crossbred) were rotationally grazed on 12-ha farmlets under three treatments. Standard pastures comprised a perennial ryegrass–clover mixture managed under contemporary practices (Std-Con). Diverse pastures were initially sown with 18 species in April 2022, and the plant species present at the final assessment in April 2025, under both contemporary (Div-Con) and regenerative (Div-Reg) management are listed in Table 1. Sward composition was determined by ten quadrat cuts per treatment that were hand‑separated by species. Species presence at the treatment level was defined as occurrence in at least one of the ten quadrat cuts taken at the final assessment.

To quantify differences in botanical diversity between standard and diverse pastures, Shannon diversity index (*H′*) was calculated for each treatment based on the number of plant species present at final assessment. Because botanical composition data were available as species presence only, the index was simplified to *H′*=ln *(S)*, where S is the number of species present [21].

Contemporary management followed current DairyNZ guidelines [22]. Regenerative farmlets received reduced mineral nitrogen (N) fertiliser inputs during Years 1–2 of the Whenua Haumanu programme [23], but no mineral N fertiliser was applied during the 2024–2025 sampling period. Regenerative management included applications of fish hydrolysate, seaweed extracts, humates, fulvic acid, and lime flour. During the sampling period, dairy regenerative farmlets received 25 L ha^− 1^ fish hydrolysate, 0.5 kg ha^−1^fulvic acid, and 5 kg ha^− 1^ seaweed extract, while sheep regenerative farmlets received 10 L ha^− 1^ fish hydrolysate, 0.5 kg ha^− 1^ fulvic acid, and 5 kg ha^− 1^ seaweed extract. These amendments supplied only trace amounts of N (< 1 kg N ha⁻¹), negligible relative to contemporary fertiliser inputs. Grazing under regenerative management involved longer rotation intervals (in average around 20 days longer) and greater post-grazing residuals (~ 500 to 1000kgDM/ha greater) compared with contemporary management.

At PCRU, four 3-ha farmlets were rotationally grazed by Romney ewes under four treatments. Standard pastures consisted of perennial ryegrass-clover swards and were managed under contemporary (Std-Con) and regenerative (Std-Reg) practices. Diverse pastures were initially sown with 19 species in April 2022 and the plant species present at the final assessment in November 2024 under both contemporary (Div‑Con) and regenerative (Div‑Reg) management, as listed in Table 1.

**Table 1:** Pasture composition, stocking rates, and nitrogen fertiliser inputs for experimental treatments in dairy and sheep systems

System	Treatments	Pasture composition*	Plant shannon diversity	Stocking rate (animals ha⁻¹)	*N* fertilisers (kg *N* ha⁻¹) ***
Dairy	Std-Con	Diploid perennial ryegrass (*Lolium perenne* L.); Tetraploid perennial ryegrass (*L. perenne* L.); White clover (large- and medium/large-leaved (*Trifolium repens* L.); Red clover (*Trifolium pratense* L.); Plantain (*Plantago lanceolata* L.) **	1.79	2.5 cows ha⁻¹	66.3
	Div-Reg	Diploid perennial ryegrass (*L. perenne* L.); Tetraploid perennial ryegrass (*L. perenne* L.); Meadow fescue (*Festuca pratensis* Huds.); Cocksfoot (*Dactylis glomerata* L.); Red clover (*T. pratense* L.); White clover (large- and medium/large-leaved (*T. repens* L.); Chicory (*Cichorium intybus* L.); Plantain (*P. lanceolata* L.)	2.20	2.0 cows ha⁻¹	None
	Div-Con	Same species composition as Div-Reg	2.20	2.0 cows ha⁻¹	53.9
Sheep	Std–Con	Diploid perennial ryegrass (*L. perenne* L.); Tetraploid perennial ryegrass (*L. perenne* L.); White clover (small- and medium/large-leaved (*T. repens* L.); Red clover (*T. pratense* L.); Plantain (*P. lanceolata* L.) **; Cocksfoot (*D. glomerata* L.) **	1.95	14 ewes ha^− 1^	38.0
	Std–Reg	Same species composition as Std–Con	1.95	12 ewes ha^− 1^	None
	Div–Con	Diploid perennial ryegrass (*L. perenne* L.); Tetraploid perennial ryegrass (*L. perenne* L.); Meadow fescue (*F. pratensis* Huds.); Cocksfoot (*D. glomerata* L.); Red clover (*T. pratense* L.); White clover (small- and medium/large-leaved, *T. repens* L.); Chicory (*C. intybus* L.); Plantain (*P. lanceolata* L.)	2.20	13 ewes ha^− 1^	37.0
	Div–Reg	Same species composition as Div–Con	2.20	12 ewes ha^− 1^	None

* Species retained at final assessment.** Species are not originally sown but present at final assessment.*** Nitrogen fertilisers inputs reflect only the paddocks sampled and the period during which sampling was conducted.

Contemporary management followed Beef + Lamb New Zealand guidelines [24], while regenerative practices mirrored those at Dairy Farm 1, with greater rotation lengths and residual post-grazing heights.

Stocking rates for both systems were determined based on the predicted pasture growth rates during late autumn and winter and to meet the treatment requirements for rotation intervals and post grazing residuals. Nitrogen fertiliser rates were determined based on treatment requirements, predicted pasture feed demand from grazing animals and an estimate of the N contributed by biological N fixation from each of the pasture treatments.

### Faecal and Soil Sampling

Faecal samples were collected directly from the rectum via manual palpation [25], ensuring that all samples were fresh and minimally exposed to environmental contamination. Samples were obtained from 10 pre-nominated cows per treatment (selected at the start of the trial to balance age and breed) and from 10 randomly selected ewes. Replacements were made for animals that became ill or were removed during the study. Ewes were selected from a uniform flock of animals balanced for age, liveweight and physiological status (all animals in same state at each sampling period).

A new sterile glove was used for each animal during sampling. Samples were transferred immediately into 5 mL sterile tubes, kept chilled during the sampling period and transport to the laboratory (within 3 h), and subsequently preserved in 1.5 mL sterile microcentrifuge tubes containing 500 µL RNAlater [26]. Samples were stored at -20 °C until analysis. All procedures were conducted in accordance with Massey Animal Ethics Committee approvals (AEC22/17, AEC22/18, AEC24/31, AEC24/47). Sampling was carried out during the third production year (2024/25) of Whenua Haumanu, with cows sampled from August 2024 to April 2025 and ewes from March to November 2024.

Soil samples were collected pre-grazing, immediately prior to livestock entry, with corresponding faecal samples collected 1–2 days later. Sampling was conducted along diagonally positioned transects using randomly selected sampling points within each paddock, while avoiding visible dung patches to minimise contamination from previous faecal inputs.

Sampling targeted Recent soils (Manawatu sandy loam, Manawatu fine sandy loam, Manawatu loamy gravel: Dystric Fluventic Eutrochrept; Rangitikei fine sandy loam: Typic Udifluvent) at Dairy Farm 1 and Pallic soils (Typic Fragiaqualf) at PCRU, classified according to both the New Zealand and USDA Soil Taxonomy systems [27–29].

Rhizosphere soil (0–7.5 cm) was collected as composite samples by combining three cores per sampling point, with thatch removed to 4 cm and root-adhering soil retained. Each composite sample was initially placed into a 5 mL sterile tube in the field, with three sampling points established along a fixed diagonal transect within each paddock (three tubes per paddock), following Taberlet et al. (2012) [30]. Samples were subsequently transferred from the 5 mL tubes into 1.5 mL sterile microcentrifuge tubes containing 500 µL RNAlater [26] at each sampling point using an ethanol-sterilised spatula. All samples were kept chilled during field collection and transport to the laboratory (within 4 h) and stored at − 20 °C until analysis.

### DNA Extraction and 16 S rRNA Gene Sequencing

Faecal and soil total DNA was extracted from 0.2 g of each sample using either the Presto™ Stool DNA extraction kit or the Presto™ Soil DNA extraction kit (Geneaid, Taiwan), following the manufacturer’s instructions. DNA purity was assessed using a NanoDrop 2000 spectrophotometer (Thermo Fisher Scientific, USA). Approximately 1 µg of quantified DNA from each sample was used to generate the sequencing library. The bacterial 16 S rRNA gene was sequenced using a 2 × 250PE version 2 chemistry on an Illumina MiSeq platform.

### Bioinformatics and Statistical Analysis

Bioinformatic analyses were performed in an Anaconda3 environment (v25.3.0), and statistical analyses were conducted using R (v4.3.3). Soil type, transect, animal breed and age were assessed as fixed effects but had no significant effect on shared taxa, alpha, or beta diversity relationships.

### Sequence Quality Control and Taxonomic Assignment

Raw sequence data were quality-checked with FastQC [31] and trimmed using an error probability cutoff of 0.01 (Phred score of 20) using SolexaQA++ [32; http://solexaqa.sourceforge.net/). Taxonomic assessments were performed using Kraken2 (v2.1.3) and Bracken (v2.9) with Greengenes 16 S database (release 13_5).

### Shared Taxa and Microbial Dominance Patterns

Shared microbial taxa were identified from species‑level Bracken outputs generated independently for soil and faecal samples. Species lists were extracted using dplyr (v1.1.4), and shared taxa were defined as those detected in both environments. For each shared species, soil and gut read counts were aggregated at the species level and used to calculate proportional contributions to each environment as Soil_contrib = soil_reads / (soil_reads + gut_reads) and Gut_contrib = gut_reads / (soil_reads + gut_reads). These complementary percentages were used to generate the two‑environment heatmap, where soil and gut panels display proportional contributions on a shared colour scale across treatments and sampling months. Heatmaps were produced using ggplot2 (v3.5.1), and the same contribution values are reported in supplementary tables.

Dominant shared taxa were identified based on aggregated read counts across all samples and time points types using the tidyverse (v2.0.0). Their temporal dynamics were visualised using ribbon plots in ggplot2 (v3.5.1), with relative abundance calculated to compare soil and gut contributions within each species across time. All read counts were normalised to relative abundance prior to analysis to account for differences in sequencing depth.

Two-way ANOVA was performed using rstatix (v0.7.2) to assess the effects of treatment, month, and their interaction on shared taxa contributions. Dominance patterns were quantified from Kraken‑derived reads for shared species. Soil and gut read counts were normalised within each environment (soil or gut) across all treatments and months, and the proportional contribution of each species was calculated. Species were classified as gut‑dominant or soil‑dominant depending on which environment contributed the higher proportion, using dplyr (v1.1.4).

### Diversity metrics and interactions

Alpha diversity (Shannon index) and beta diversity (Bray-Curtis dissimilarity) were calculated directly from Bracken species abundance files using the diversity scripts in KrakenTools (v1.2) [33, 34]. These scripts internally convert Bracken counts to relative abundances by dividing each taxon’s count by the total sample count prior to computing diversity metrics, thereby accounting for differences in sequencing depth across samples. Boxplots were generated using the ggplot2 (v3.5.1) to visualise variation in Shannon index across treatments and sampling time points.

Linear mixed-effects models (LMMs) were fitted using the lme4 (v1.1.36), lmerTest (v3.1.3), emmeans (v1.11.2), and dplyr (v1.1.4) to examine interactions in alpha diversity between source (soil vs. gut) and treatment [35]. Models included source, treatment, and their interaction (source × treatment) as fixed effects, with month as a random effect to account for sampling at multiple time points across rotational paddocks within treatments. Reference levels were Std-Con for treatment and gut for source, thereby model estimates represent differences relative to these references, with t-values indicating effect strength and p-values indicating statistical significance. Estimated marginal means and pairwise contrasts between soil and gut were calculated. Model assumptions were checked using the Shapiro-Wilk test for normality and residual plots for homogeneity of variance.

Spearman rank correlations between gut and soil alpha diversity were calculated per treatment by pooling months. Spearman correlation coefficients (*r*_*s*_) were not calculated separately per month due to limited soil replicates per treatment (*n* = 3). The strength of correlations was described based on the absolute value of correlation coefficients [36].

Procrustes analysis was used to compare soil and gut beta diversity by superimposing principal coordinate analysis (PCoA) ordinations of Bray-Curtis dissimilarity matrices across treatments and months, to evaluate the similarity of community structures. Procrustes correlations (procrustes 𝑟) and p-values (999 permutations) were calculated using vegan (v2.6.10).

## Results

### Shared Bacteria Species Between Soil and gut

**Fig. 1:**
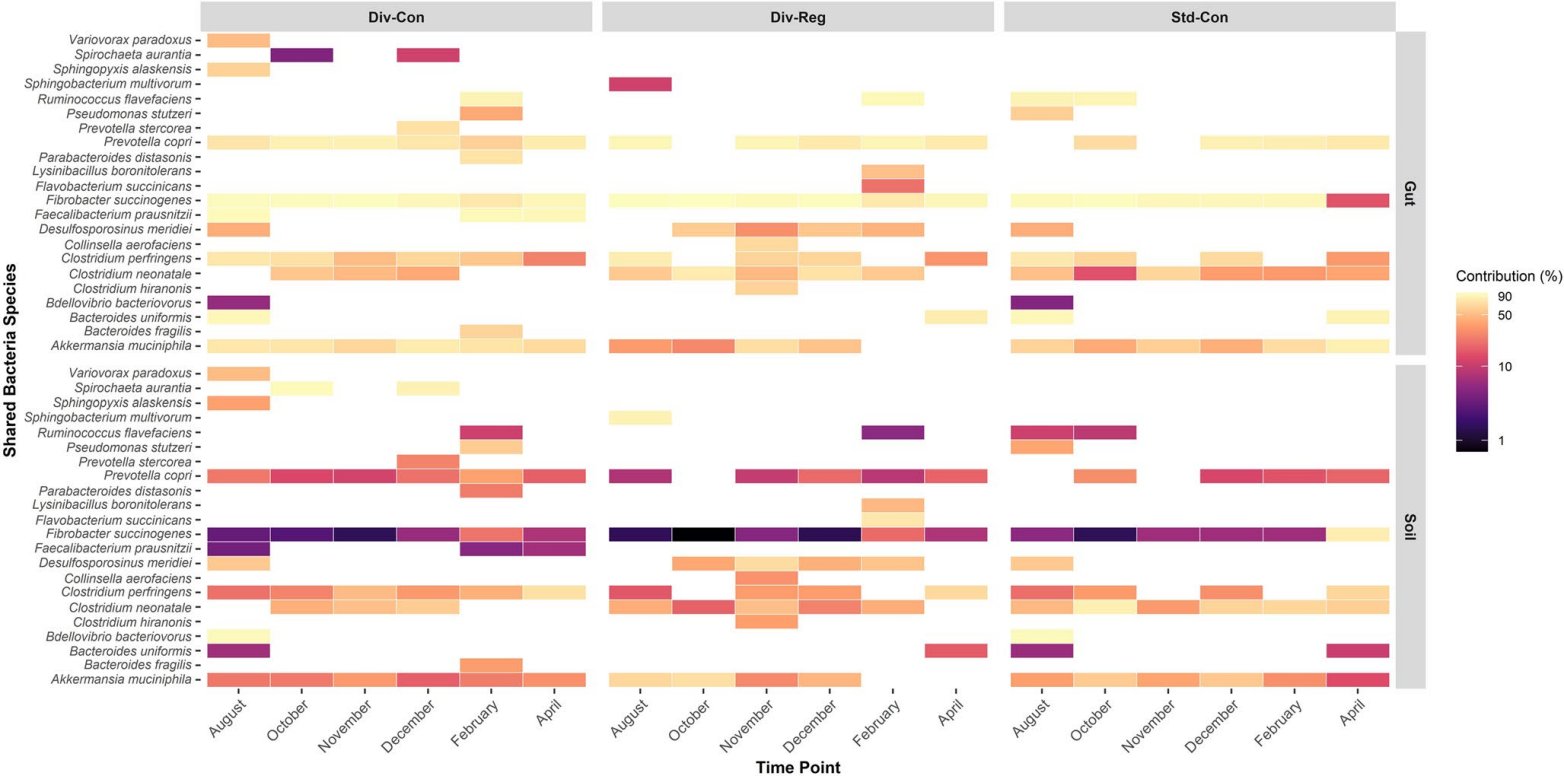
Heatmap showing the temporal soil–gut contributions of bacterial species shared between soil and the cattle gut across three treatments (Std-Con, Div-Reg, and Div-Con) from August 2024 to April 2025. For each shared species, soil and gut contributions were calculated as the percentage of total reads (soil + gut) attributed to each environment. Rows represent shared taxa and columns represent monthly sampling time points within each treatment. Colour intensity (magma scale) reflects the percentage contribution to each environment, with lighter colours indicating higher contributions and darker colours indicating lower contributions

**Fig. 2:**
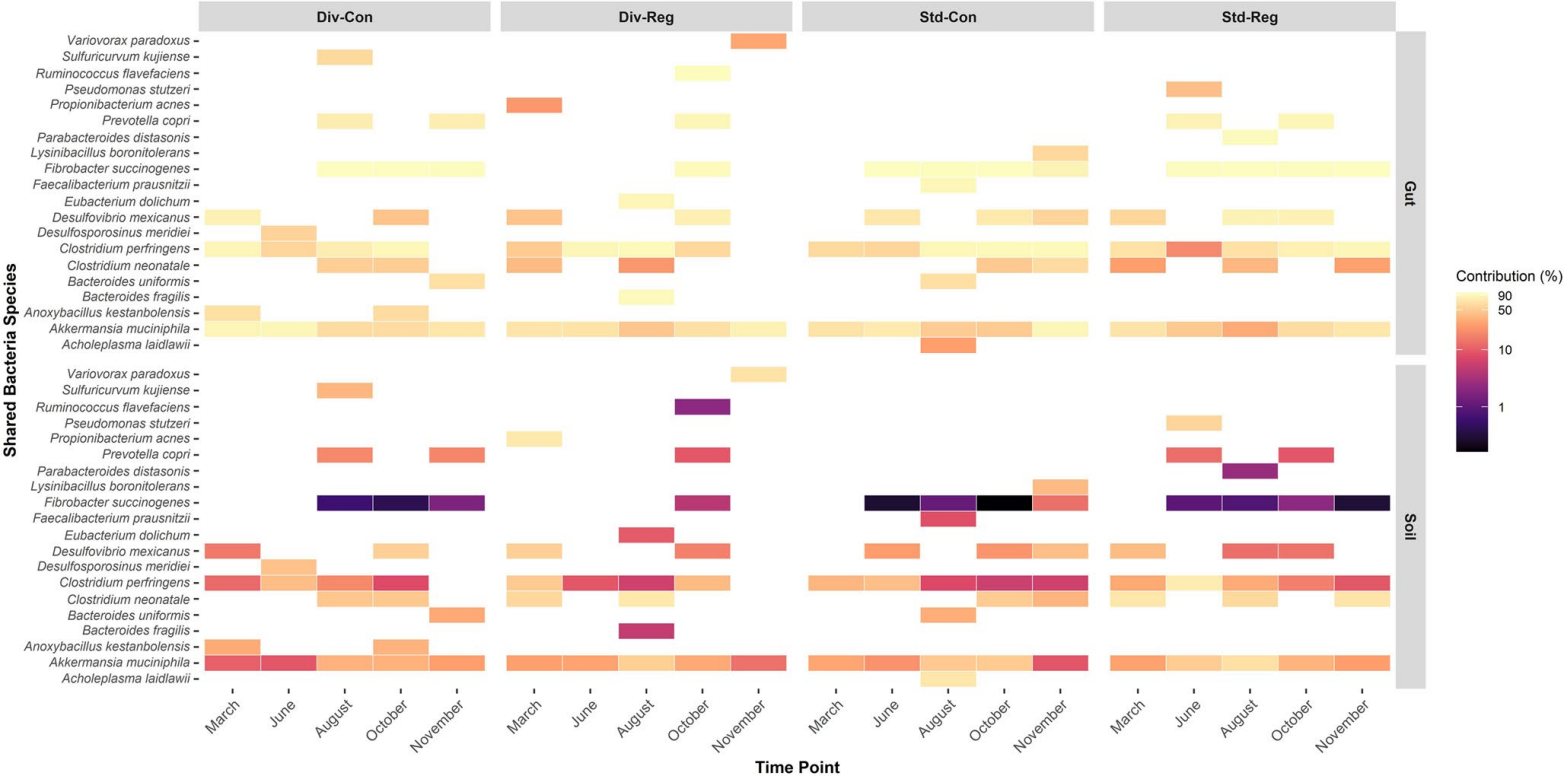
Heatmap showing the temporal soil–gut contributions of bacterial species shared between soil and the sheep gut across four treatments (Std‑Con, Std‑Reg, Div‑Con, Div‑Reg) from March to November 2024. For each shared species, soil and gut contributions were calculated as the percentage of total reads (soil + gut) attributed to each environment. Rows represent shared taxa and columns represent monthly sampling time points within each treatment. Colour intensity (magma scale) reflects the percentage contribution to each environment, with lighter colours indicating higher contributions and darker colours indicating lower contributions

**Fig. 3:**
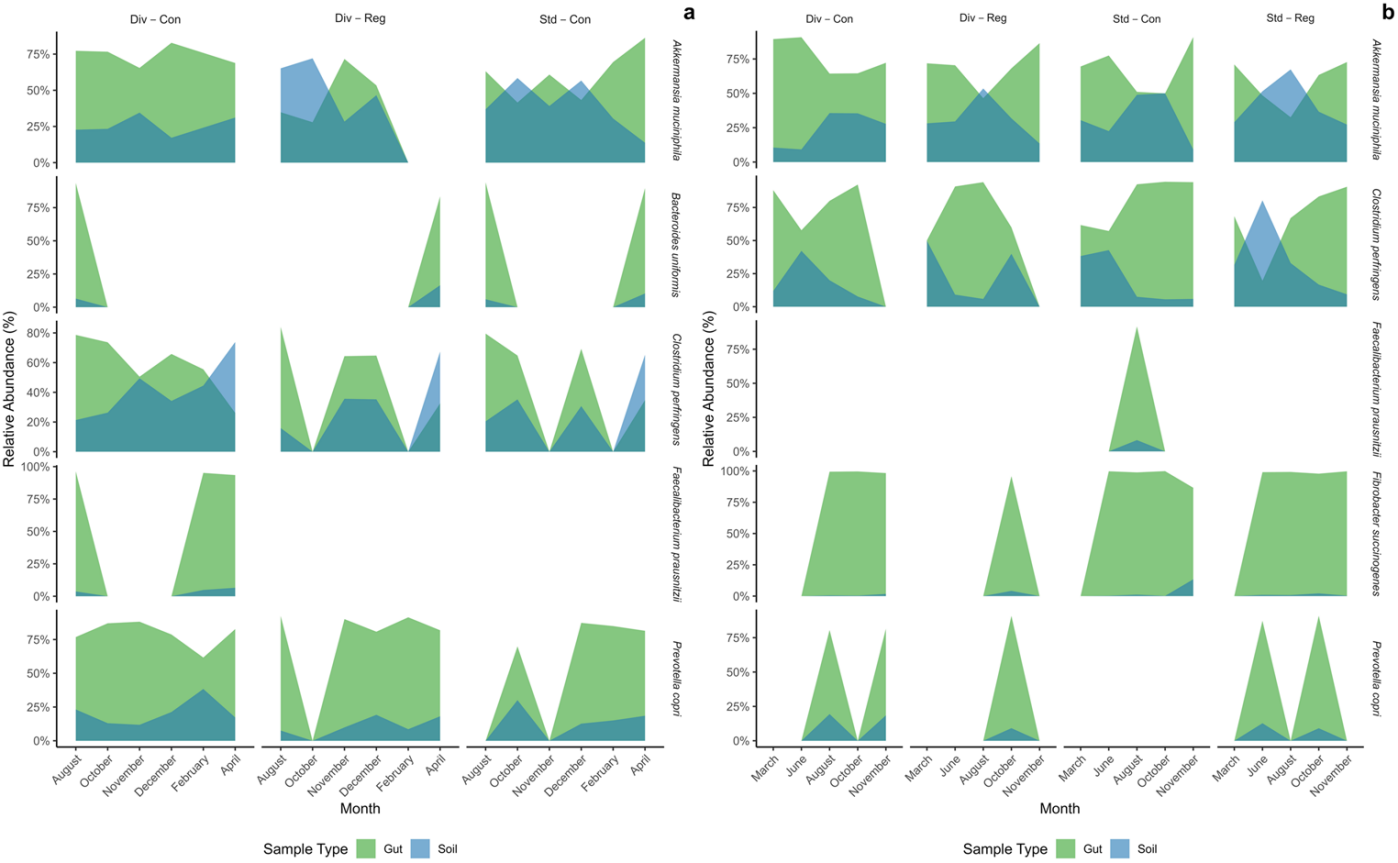
Temporal variation in dominant shared bacterial species based on relative abundance across treatments: (**a**) soil–cattle gut from August 2024 to April 2025 (Std-Con, Div-Reg, Div-Con), and (**b**) soil–sheep gut from March to November 2024 (Div-Con, Div-Reg, Std-Con, Std-Reg). Relative abundance was calculated for each species within treatment and month to compare its distribution between soil and gut samples. Green ribbons represent gut samples and blue ribbons represent soil samples

Heatmaps show the relative abundance of bacterial species shared between soil and cattle/sheep gut across treatments and sampling months (Figs. 1 and 2). Temporal patterns of the dominant shared taxa are presented in Fig. 3. In the dairy system (Fig. 3a), *Prevotella copri*, *Faecalibacterium prausnitzii*, *Akkermansia muciniphila*, *Bacteroides uniformis*, and *Clostridium perfringens* consistently appeared among the most abundant shared taxa across months, with clear differences in their relative contributions to soil and gut communities over time. In the sheep system (Fig. 3b), *C. perfringens*, *A. muciniphila*, *Fibrobacter succinogenes*, *P. copri*, and *F. prausnitzii* dominated across treatments and sampling months, showing distinct temporal shifts in their relative abundance between soil and gut samples.

### Soil and Cattle gut

For the Std-Con treatment grazed by cattle, *(A) muciniphila* contributed more (60–85%) to the gut community than to soil across most of the study period, except for October (58.4%) and December (56.7%), when its contribution was greater in soil. *Clostridium perfringens* remained gut-dominant but was not detected in either compartment in November or February. *Prevotella copri* was continuously shared between soil and gut from October to April, consistently showing greater contributions in the gut, whereas *(B) uniformis* was only detected in August and April and was strongly enriched in the gut (89–94%). *Clostridium neonatale* was generally soil-dominant, except in August and October when its contribution was higher in the gut, and *F. succinogenes* maintained consistently greater contributions in the gut across all sampling months. Additionally, *Pseudomonas stutzeri* and *Ruminococcus flavefaciens*, exhibited higher contributions in the gut than in soil, whereas the predatory bacterium *Bdellovibrio bacteriovorus* contributed more strongly to the soil community (Fig. 1, Table S1).

In Div-Reg, *A. muciniphila* contributed more to the soil community in August (65.2%) and October (72.1%), but its greater contribution shifted to the gut from November (71.6%) to December (53.4%), after which it was no longer detected as a shared soil–gut taxon. *Clostridium perfringens*,* C. neonatale*,* P. copri*, and *F. succinogenes* were also shared between soil and gut and generally showed greater proportions in the gut, whereas *Sphingobacterium multivorum*,* F. succinicans*, and *Lysinibacillus boronitolerans* contributed more to the soil (Fig. 1, Table S1).

In Div-Con, *A. muciniphila* and *P. copri* were consistently present throughout the study period, with higher relative contributions in the gut. *Clostridium perfringens* also exhibited higher gut contributions in most months, except in April, showing greater abundance in the soil (73.9%). *Faecalibacterium prausnitzii* appeared as a shared taxon from February (95.2%) to April (93.4%), predominantly contributing to the gut. *Bacteroides uniformis* was only detected in August (93.5%) in gut samples, whereas *C. neonatale* only appeared as a shared taxon in October but shifted to soil only detection from November to December. In addition, *Sphingopyxis alaskensis*, *Prevotella stercorea*, and *Parabacteroides distasonis* contributed mainly to the gut, while *Variovorax paradoxus*, *Spirochaeta aurantia*, and *Pseudomonas stutzeri* contributed predominantly to the soil (Fig. 1, Table S1).

The contributions of soil-gut shared taxa were not significantly affected by treatment, sampling month, or their interaction (*p* > 0.8 for all comparisons).

### Soil and Sheep gut

In Std-Con, *A. muciniphila* and *C. perfringens* consistently appeared as shared taxa between soil and the sheep gut, typically showing greater contributions in the gut. However, in October, both *A. muciniphila* (~ 50%) and *C. neonatale* (~ 51%) contributed equally to soil and gut communities.

*Faecalibacterium prausnitzii* was identified as a shared, gut-enriched taxon only in August, contributing 91.7% to the gut. *Fibrobacter succinogenes* appeared as a shared, gut-enriched taxon from June onward, and *B. uniformis* was detected only in August, contributing predominantly to the gut (66.4%) (Fig. 2, Table S2).

In Std-Reg, *A. muciniphila* and *C. perfringens* shifted from gut dominance to soil dominance between June and August, whereas *F. succinogenes* and *P. copri* remained enriched in the gut. *Clostridium neonatale* and *P. stutzeri* appeared intermittently as shared taxa but contributed more substantially to the soil community (Fig. 2, Table S2).

In Div-Con, *A. muciniphila*, *C. perfringens*, *C. neonatale*, *F. succinogenes*, and *P. copri* were shared between soil and gut, generally contributing more to the gut. *Bacteroides uniformis* appeared only in November (68.2%), with a high gut contribution to the gut (Fig. 2 and Table S2).

Similarly, in Div-Reg, *A. muciniphila*, *C. perfringens*, and *C. neonatale* were detected as shared taxa, with some variation in their relative contributions to soil and gut across months. Notably, in August, *Eubacterium dolichum* was shared between soil and gut but contributed predominantly to the gut (90.2%), and *Bacteroides fragilis* also appeared as a gut-enriched taxon (94.9%), whereas *Desulfovibrio mexicanus* contributed more than 45% to the soil community in March (Fig. 2, Table S2).

Consistent with the cattle system, statistical analysis indicated that treatment, sampling month, and their interaction did not significantly affect the relative contributions of soil-gut shared taxa (*p* > 0.3 for all comparisons).

### Microbiome Dominance Patterns

**Fig. 4:**
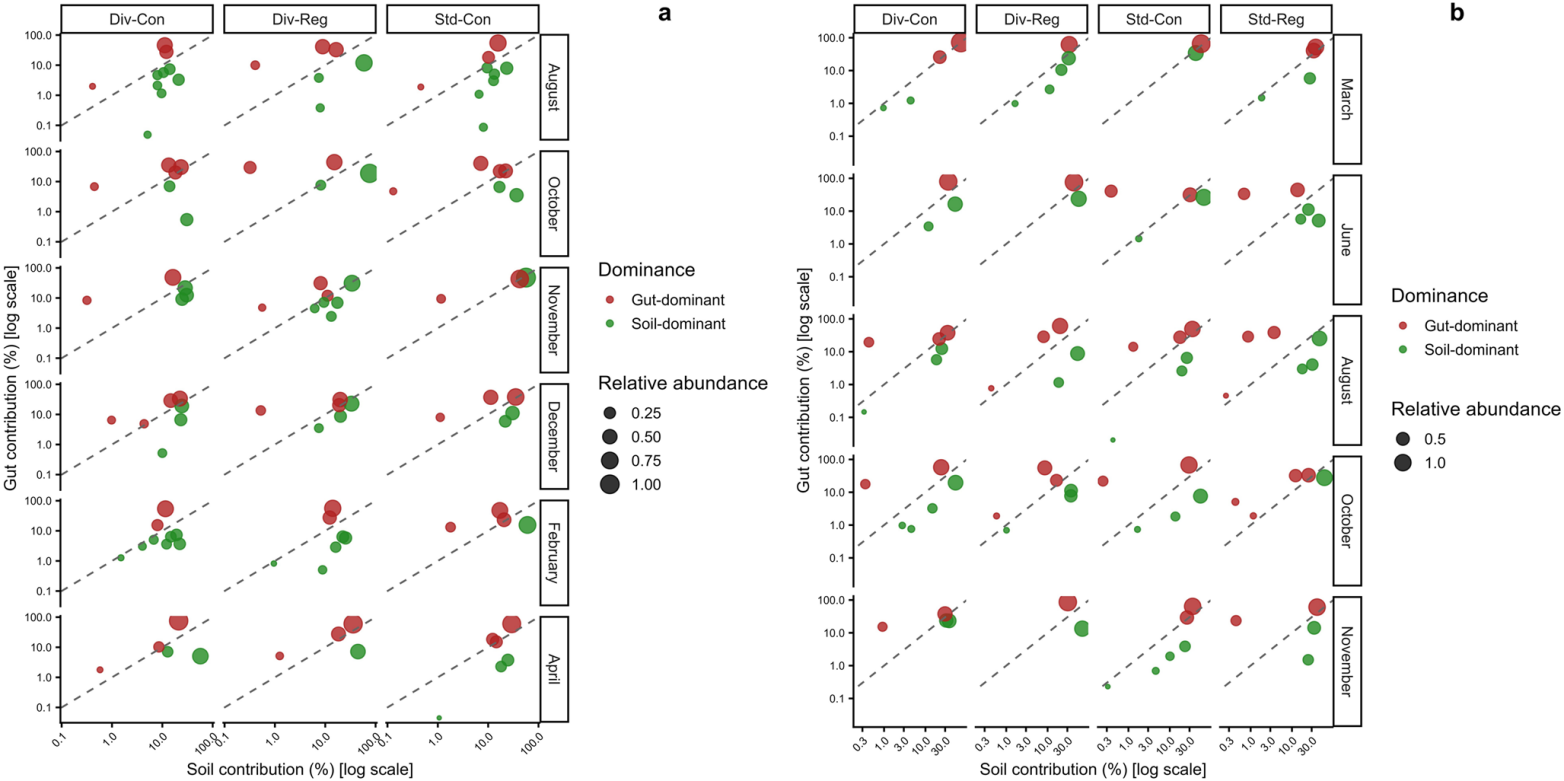
Dominance patterns of shared bacterial species between soil and gut microbiomes across treatments in (**a**) cattle (Std-Con, Div-Reg, Div-Con) and (**b**) sheep (Std-Con, Std-Reg, Div-Reg, Div-Con). Scatter plots show the percentage contributions of shared bacterial taxa in soil (x‑axis) and gut (y‑axis) across treatments and time points. Point size reflects total relative abundance, and colour indicates dominance (red = gut‑dominant; green = soil‑dominant). The dashed 1:1 line marks equal contribution, with points above or below the line representing gut‑ or soil‑dominant taxa, respectively

Figure 4 shows the 1:1 diagonal line (dashed black line) separating gut‑dominant taxa (points above the line, red) from soil‑dominant taxa (points below the line, green) across all treatments and sampling months for cattle (Fig. 4a) and sheep (Fig. 4b).

In the cattle system, soil‑dominant taxa were few and clustered tightly around the 1:1 line, indicating only weak soil dominance. In contrast, gut‑dominant taxa showed larger deviations above the line, reflecting a stronger and more consistent gut contribution. Dominance patterns were stable across months, and no clear treatment‑driven differences were evident.

In the sheep system, gut‑dominant taxa formed a more distinct and pronounced pattern above the 1:1 line, indicating a stronger gut influence compared with cattle. Soil‑dominant taxa were more frequently observed under regenerative treatments (Std‑Reg, Div‑Reg) than under contemporary treatments (Std‑Con, Div‑Con), although these taxa also remained close to the 1:1 line, suggesting weak soil dominance. As in cattle, overall clustering patterns were consistent across treatments and sampling points.

### Diversity and Interactions

**Fig. 5:**
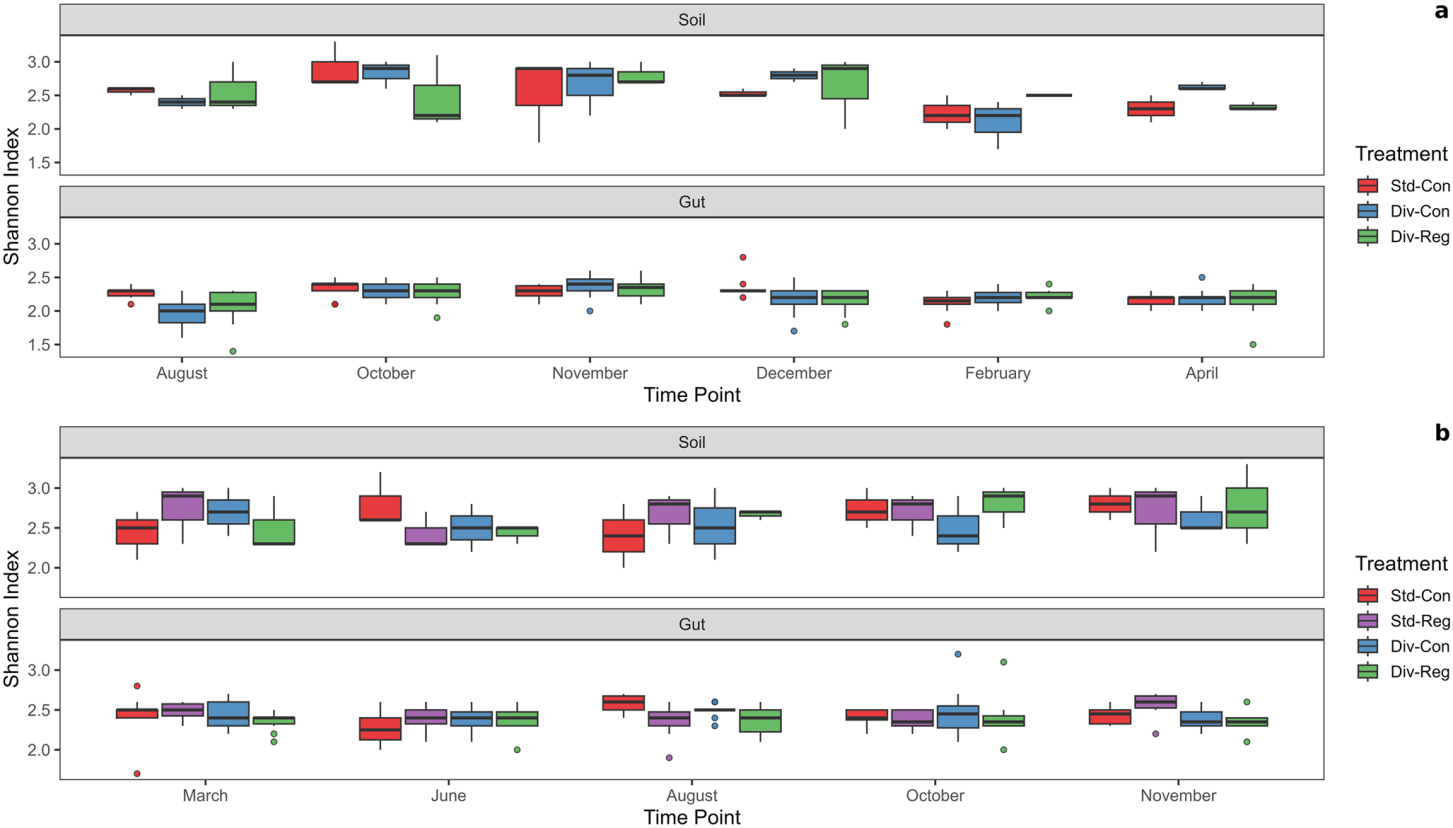
Temporal variation in bacterial alpha diversity (Shannon index) in (**a**) Recent soils (top panel) and cattle gut (bottom panel) across grazing treatments from August 2024 to April 2025 and (**b**) Pallic soil (top panel) and sheep gut (bottom panel) across treatments from March-November 2024. Boxplots show the distribution of shannon diversity at each time point for Std-Con (red), Std-Reg (purple), Div-Con (blue), and Div-Reg (green)

Shannon index analysis revealed that soil microbiome diversity was consistently greater than gut microbiome diversity for both cattle and sheep systems, regardless of treatment or sampling time. Gut diversity remained stable with little variation, while soil diversity was always greater (Fig. 5a, b).

Cattle and sheep had significant differences between their gut microbiomes (cattle: *p* = 0.001; sheep: *p* = 0.002) (Table 2). However, there was no effect of treatment or interactions, indicating the soil-gut diversity gap persisted across all experimental conditions (cattle: all *p* > 0.16; sheep: all *p* > 0.4) (Table 2). Pairwise contrasts using emmeans identified significantly greater soil diversity within each treatment (Table 2). Random effect estimates for Month were small and overlapped zero in both systems (cattle: variance = 0.0082, SD = 0.09; sheep: variance = 0.00085, SD = 0.03), indicating minor monthly variation in alpha diversity (Table 2).

**Table 2:** Linear mixed-effects model results for Shannon index alpha diversity (soil vs. gut)

Section		Effect / Treatment	Effect Size (Estimate)	Standard Error	t value	*p*-value	
Cattle system: Recent soils vs. cattle gut alpha diversity							
Fixed Effects		Intercept (Gut/Std-Con)	2.26	0.05	47.7	0.001	
		Source (soil vs. gut)	0.26	0.06	4.27	0.001	
		Treatment: Div-Con	-0.06	0.04	-1.4	0.165	
		Treatment: Div-Reg	-0.06	0.04	-1.52	0.130	
		Source × Treatment (Soil-gut difference x Div-Con)	0.12	0.08	1.41	0.162	
		Source × Treatment (Soil-gut difference x Div-Reg)	0.10	0.08	1.20	0.230	
Pairwise Contrasts (emmeans)		Std-Con: Gut – Soil	-0.256	0.06	-4.27	0.0001	
		Div-Con: Gut – Soil	-0.375	0.06	-6.26	0.0011	
		Div-Reg: Gut – Soil	-0.358	0.06	-5.97	0.0012	
Random Effect (Month): Variance = 0.0082, SD = 0.09							
Sheep system: Pallic soil vs. sheep gut alpha diversity							
Fixed Effects	Intercept (gut/Std-Con)		2.42	0.036	67.70		0.001
	Source (soil vs. gut)		0.210	0.066	3.20		0.002
	Treatment: Div-Con		0.018	0.047	0.39		0.699
	Treatment: Std-Reg		-0.050	0.047	-1.06		0.289
	Treatment: Std-Reg		0.002	0.047	0.05		0.962
	Source × Treatment (Soil-gut difference x Div-Con)		-0.078	0.093	-0.84		0.403
	Source × Treatment (Soil-gut difference x Div-Reg)		0.050	0.093	0.54		0.592
	Source × Treatment (Soil-gut difference x Std-Reg)		0.011	0.093	0.12		0.906
Pairwise Contrasts (emmeans)	Div-Con: Gut – Soil		-0.133	0.066	-2.01		0.0455
	Div-Reg: Gut – Soil		-0.261	0.066	-3.95		0.0001
	Std-Con: Gut – Soil		-0.211	0.066	-3.20		0.0016
	Std-Reg: Gut – Soil		-0.222	0.066	-3.36		0.0009

Random Effect (Month): Variance = 0.00085, SD = 0.03

Spearman’s rank correlations between soil and gut alpha diversities varied across treatments in both sheep and cattle, with none reaching statistical significance (Table 3). In cattle, weak positive correlations were observed in Div-Con (*r*_*s*_ = 0.31, *p* = 0.56) and Div-Reg (*r*_*s*_ = 0.31, *p* = 0.7), while Std-Con exhibited a moderately strong positive correlation (*r*_*s*_ = 0.67, *p* = 0.15) (Table 3). In sheep, the correlations ranged from very weakly positive in Div-Con (*r*_*s*_ = 0.05, *p* = 0.94) to moderately strong positive in Std-Reg (*r*_*s*_ = 0.6, *p* = 0.35), with Div-Reg showing a weak positive correlation (*r*_*s*_ = 0.32, *p* = 0.60) and Std-Con a moderately strong negative correlation (*r*_*s*_ = -0.7, *p* = 0.23) (Table 3).

**Table 3:** Spearman’s rank correlations between soil and gut alpha diversity

Treatment	Spearman ρ	*p*-value
Cattle system		
Div-Con	0.31	0.56
Div-Reg	0.32	0.7
Std-Con	0.67	0.15
Sheep system		
Div-Con	0.05	0.94
Div-Reg	0.31	0.6
Std-Con	-0.7	0.23
Std-Reg	0.6	0.35

Procrustes analysis was used to compare beta diversity of soil and gut microbiome across treatments and sampling months in cattle and sheep systems (Table 4). Procrustes correlations (procrustes 𝑟) varied across months and treatments in both systems (cattle: 0.51–0.99; sheep: 0.57–0.98), but none were statistically significant. In cattle, the highest correlation was observed in Div-Reg in April (procrustes 𝑟 = 0.99, *p* = 0.16) and the lowest in Div-Con in April (procrustes 𝑟 = 0.51, *p* = 0.8). In sheep, the highest correlation occurred in Std-Con in August (procrustes 𝑟 = 0.98, *p* = 0.16), and the lowest in Div-Con in October (procrustes 𝑟 = 0.57, *p* = 0.83).

**Table 4:** Procrustes correlations (r) between soil and gut microbiome beta diversity across months and grazing treatments in cattle and sheep systems

Cattles grazing system					Sheep grazing system				
Month	Treatment	Procrustes Correlation (procrustes *r*)		*p* value	Month	Treatment		Procrustes Correlation (procrustes *r*)	*p* value
August	Std-Con	0.63		0.55	March	Std-Con		0.84	0.83
	Div-Con	0.74		0.46		Std-Reg		0.68	0.81
	Div-Reg	0.64		0.54		Div-Con		0.92	0.66
October	Std-Con	0.77		0.67		Div-Reg		0.74	0.82
	Div-Con	0.85		0.50	June	Std-Con		0.68	0.66
	Div-Reg	0.76		0.50		Std-Reg		0.86	0.50
November	Std-Con	0.85		0.50		Div-Con		0.72	0.62
	Div-Con	0.98		0.67		Div-Reg		0.80	0.58
	Div-Reg	0.77		0.83	August	Std-Con		0.98	0.16
December	Std-Con	0.86		0.50		Std-Reg		0.78	0.82
	Div-Con	0.76		0.66		Div-Con		0.67	0.66
	Div-Reg	0.88		0.50		Div-Reg		0.92	0.12
February	Std-Con	0.85		0.90	October	Std-Con		0.93	0.33
	Div-Con	0.91		0.50		Std-Reg		0.83	0.16
	Div-Reg	0.83		0.80		Div-Con		0.57	0.83
April	Std-Con	0.8		0.50		Div-Reg		0.70	0.64
	Div-Con	0.51		0.8	November	Std-Con		0.93	0.83
	Div-Reg	0.99		0.16		Std-Reg		0.63	0.12
						Div-Con		0.91	0.82
							Div-Reg	0.86	0.66

## Discussion

This study provides insights into soil-gut microbial interactions in cattle and sheep grazing systems across different pasture management practices. It presents a consistent sharing of specific bacterial taxa between soil and gut microbiomes, with these shared taxa typically more abundant in the gut than in soil. In contrast, soil harboured a greater overall bacterial diversity than the gut, and correlations in diversity metrics between soil and gut were weak to moderate and non-significant. Importantly, none of these measures (shared taxa contributions, dominance patterns, and diversity metrics) were strongly influenced by pasture diversity, management practice, or their interaction, indicating that soil–gut microbiome linkages are resilient to the applied treatments.

### Shared Taxa and Functional Roles

The most abundant shared taxa between soil and gut microbiomes across treatments and sampling time points were *P. copri*, *F. prausnitzii*, *(A) muciniphila*, *(B) uniformis*, *(C) perfringens* and *F. succinogenes* (Fig. 4). These taxa exemplify soil–gut microbial connectivity characteristic of New Zealand pastoral systems, where ingested soil during grazing contributes 2.6–8% of the gut microbiome [4].

*Prevotella copri* was the most abundant shared taxon in cattle, consistent with its well-established role as a key fibre-degrading bacterium in the rumen [37, 38]. *Prevotella* species possess extensive polysaccharide utilisation loci (PULs) encoding carbohydrate-active enzymes specialised in degrading non-cellulosic plant fibres and enhancing branched-chain amino acid synthesis, contributing to muscle growth and productivity [38, 39]. Its consistent gut dominance across treatments reflects gut niche specialisation rather than variations in pasture management, highlighting the specialised functional role of *Prevotella* in fibre degradation within the anaerobic, nutrient-rich gut environment [37, 38].

*Akkermansia muciniphila* showed dynamic temporal variation in dominance patterns, shifting between soil and gut dominance across sampling months and treatments. In the gut, *A. muciniphila* plays an ecologically important role as a mucin-degrading bacterium [40, 41] and utilises mucin as its sole carbon (C), N, and energy source, producing acetate and propionate while releasing mucin-derived oligosaccharides that support cross-feeding by other beneficial bacteria including *F. prausnitzii* [42, 43]. The presence of *A. muciniphila* in soil samples indicates either environmental persistence or functional versatility. However, current literature provides no direct evidence for survival of *A. muciniphila* in the soil environment [39, 44]. Thus, this finding remains uncertain and requires further investigation. Future studies incorporating viability assays, controlled soil microcosm experiments, and activity-based analyses would help determine whether it is metabolically active in soil or only transiently present.

A pronounced temporal shift of *A. muciniphila* abundance was evident in the Div‑Reg treatment for the cattle system, transitioning from soil (65–72%) to gut dominance (53–72%) between August and December. This pattern is consistent with post-winter pasture growth reducing soil ingestion [45], potentially enhancing gut barrier maintenance via *F. prausnitzii* cross-feeding under improved spring nutrition [46].

*Bacteroides uniformis* appeared sporadically but with a consistently high contribution to the gut population, indicating selective colonisation rather than continuous exchange. *Bacteroides uniformis* is a key starch degrader and producer of short-chain fatty acids in the gut, contributing to host energy metabolism and immune modulation [47]. The intermittent detection pattern for *B. uniformis* may reflect seasonal variation in pasture starch content or host dietary preferences that create favourable conditions for establishment.

The functional roles of *F. prausnitzii* and *C. perfringens* as shared taxa are also notable. *F. prausnitzii*, a major butyrate‑producing bacterium and marker of intestinal health [48], appeared as a gut‑dominant shared taxon specifically in contemporary treatments (Div‑Con in cattle and Std-Con in sheep) while being absent from regenerative treatments. This management-specific pattern may reflect higher nitrogen fertiliser rates typical of contemporary management favouring *Faecalibacterium* growth under strictly anaerobic rumen conditions [22, 49]. Conversely, while *C. perfringens is* often associated with pathogenicity, is also a normal constituent of ruminant gut microbiomes generally at low levels, and its shifts in soil dominance may reflect faecal deposition and the persistence of spores in soil [50].

*Fibrobacter succinogenes* showed consistently greater gut contributions across treatments, with detection in Std-Con in sheep systems increasing from June onward (Table S2), which may reflect seasonal cellulose availability peaks. This obligate anaerobe dominates rumen cellulose degradation, producing succinate [51] but cannot proliferate under aerobic soil conditions [52]. Its strong gut enrichment supports observed host filtering effect.

Importantly, non-significant differences among pasture diversity, management practice, and sampling time points suggest that core soil-gut microbial interactions may be relatively stable and resilient to variations in management practices or pasture diversity within the context of this study. However, the sporadic appearance of specific taxa such as *Sphingobacterium multivorum*, *Lysinibacillus boronitolerans*, and *Parabacteroides distasonis* in diverse pasture treatments indicate that pasture composition may broaden the range of shared species, even if it does not change the dominance patterns of the core shared taxa [53].

### Directionality of Soil–gut Microbial Dominance

In both the cattle and sheep systems, gut‑dominant taxa were consistently more abundant than soil‑dominant taxa, and this pattern was similar across all treatments and sampling time points. This indicates that microbial exchange in these grazing systems is not a balanced two‑way process and may be biased from gut to soil, with the ruminant gut playing a major role in shaping the shared microbiome [4]. However, this pattern does not exclusively indicate gut‑to‑soil transmission, as soil‑derived taxa may enter the gut at low abundance and be selectively retained in the gut by host filtering.

Several processes may contribute to this pattern. Grazing ruminants deposit large amounts of faeces onto pasture, adding very high numbers of gut microbes in concentrated locations to the soil over time, whereas only relatively small microbial loads are ingested with soil particles or on the surfaces of the forages during grazing. Although soil in this study was collected before the grazing events, past faecal deposition on these paddocks may have contributed to this gut-soil microbial distribution pattern. Many common rumen and intestinal bacteria are adapted to anaerobic conditions in the gut and are unable to grow in well‑aerated soils [54]. Their cells may become dormant or die in soil, but their DNA can still be detected by 16 S rRNA sequencing, which can inflate their presence in soil. In addition, the gut environment can act as a strong filter, through factors such as pH, anaerobiosis, bile, immune defences and specific nutrient inputs, which will favour the establishment of gut commensals and limit colonisation by microbes originating from soil [55, 56].

The occasional appearance of soil‑dominant taxa in gut samples indicates that under some conditions, microbes from soil can enter and temporarily proliferate in the gut community. This pattern was most apparent in terms of frequency under regenerative pasture management, particularly in the sheep farmlets. Regenerative systems typically use longer rest periods between grazings and leave greater post‑grazing pasture residuals which can increase the amount of soil and organic debris adhering to lower leaves and stems [57]. Sheep tend to graze closer to the soil surface than cattle and selectively removing fine green material from these lower layers, further increasing the chance of ingesting soil particles and associated microbes [58]. However, the low abundance and rare detection of these taxa may indicate that, even under such conditions, microbes from soil make only a small and intermittent contribution to the gut community compared with the persistent influence of gut taxa.

### Soil-gut Diversity Relationships

Soil consistently exhibited greater alpha diversity than gut microbiomes across cattle and sheep systems, reflecting fundamental ecological differences between these habitats (Fig. 5; Table 2). Soil supports extremely diverse microbial communities as it contains many microhabitats, a wide range of C sources and strong spatial and chemical heterogeneity [59]. In contrast, the gut is a more selective, functionally specialised, environment in which a narrower subset of microbes adapted to anaerobic conditions [60].

Treatment-independent soil–gut diversity differences suggest that variation in diversity was not strongly driven by pasture species compositions or management practices. Although diverse pastures had higher plant Shannon diversity than standard pastures (Table 1), we did not detect corresponding differences in soil or gut Shannon diversity between these treatments, and soil–gut diversity correlations were weak and non‑significant across all systems. This indicates that, within the timeframe of this study, the influence of pasture species diversity on soil or gut microbial diversity remains inconclusive. Instead, other factors such as host genetics, immune function, gut physiology, and core dietary components shared across treatments may play a greater role in shaping gut microbial diversity than pasture composition or management approach [61]. The absence of statistically significant correlations between soil and gut alpha diversity across treatments is informative (Table 3). These weak or negative correlations indicate that soil is not the main source driving changes in the gut microbiome, and the gut community in ruminants is mainly governed by processes inside the animal. From this we hypothesise that gut conditions act as strong filters, inhibiting most soil microbes from colonising the gut [53].

Procrustes analysis comparing beta diversity between soil and gut microbiomes revealed a wide range of correlation strengths across sampling points and treatments (Table 4). The strongest correlations occurred only at a few time points (Div‑Reg cattle in April and Std‑Con sheep in August) (Table 4), indicating occasional shifts in soil and gut communities may synchronised changes in pasture growth stage and soil conditions. However, the absence of significant effect for these high correlations reflects the small sample size from soils and the large natural variability in both soil and gut communities in complex grazing systems.

## Conclusions and Future Perspectives

This study demonstrates the presence of soil–gut microbial connectivity in grazing cattle and sheep systems, although the shared microbial taxa are typically more abundant in the gut, and soil consistently supports higher overall diversity. The absence of strong associations between soil and gut diversity metrics suggests that gut microbial communities are primarily structured by host-specific ecological processes and internal selection pressures, rather than by continuous microbial input from soil or pasture management practices. The consistency of these patterns across treatments indicates that core soil–gut interactions may be relatively stable within pastoral environments. However, greater pasture diversity may modestly broaden the range of shared taxa.

A further limitation of this study is that fungal communities were not assessed, as the sequencing approach targeted the bacterial 16 S rRNA gene and therefore captured only bacterial taxa. Previous studies in grazing systems suggest that fungi may persist longer in the environment and be more readily transferred across the soil–plant–rumen continuum than bacteria [4]. Therefore, the exclusion of fungal communities may limit a comprehensive understanding of microbial connectivity and its ecological implications in pastoral systems.

Future studies should aim to address key methodological limitations by moving beyond 16 S rRNA gene sequencing, which cannot distinguish active from inactive cells, to include RNA-based approaches, metatranscriptomics, and analyses of other microbial groups such as fungi, archaea, protozoa, and viruses. Such approaches would allow clearer identification of metabolically active taxa and their functional roles in both soil and animal systems.

Long-term, integrative studies that track individual animals alongside the specific grazing areas they utilise, combined with measurements of nutrient cycling, plant performance, animal productivity, health, and immune function, and supported by functional genomic analyses, will be essential for developing management strategies that leverage soil–gut microbial linkages to enhance the sustainability and resilience of pastoral agriculture.

## Supplementary Information

Below is the link to the electronic supplementary material.

Supplementary Material 1 (DOCX 38.7 KB)

Supplementary Material 2 (DOCX 35.0 KB)

Supplementary Material 3 (CSV 10.4 KB)

## Data Availability

Raw 16 S rRNA gene sequencing reads for the bulk cattle and sheep gut microbiomes have been deposited in the European Nucleotide Archive (ENA) under the previous project accession PRJEB105277. Selected accession numbers (*n*=323) for sequence reads associated with this study are provided in the Supplementary Information (Supplementary Material 3: Table S3: Related accessions for gut microbiome reads included in this study) ( [https://www.ebi.ac.uk/ena/browser/view/PRJEB105277](https:/www.ebi.ac.uk/ena/browser/view/PRJEB105277). The corresponding raw 16 S rRNA gene sequencing reads for the soil microbiome have been deposited in the ENA under the project accession PRJEB108514 ( [https://www.ebi.ac.uk/ena/browser/view/PRJEB108514](https:/www.ebi.ac.uk/ena/browser/view/PRJEB108514) ).

## Declarations

### Use of Large Language Models (LLMs)

During the preparation of first draft, the first author used ChatGPT (OpenAI), to assist with language editing and improve clarity. Following the use of this tool, the author(s) reviewed and edited the content as necessary and take full responsibility for the content of the published article.
