# Supplementary material for: Host Filtering Shapes the Soil–gut Microbiome Linkages in Pastoral Systems
Source: Microb Ecol. 2026 May 21;89(1):146. doi: 10.1007/s00248-026-02791-6 (PMC13369704; doi:10.1007/s00248-026-02791-6)
Supplement: Supplementary file 1 — Supplementary Material 1 (DOCX 38.7 KB) [file 248_2026_2791_MOESM1_ESM.docx]

**Host filtering shapes the soil–gut microbiome linkages in pastoral systems**

Upulika Jayaneththi ^a,c^*, Nicholas W. Sneddon ^a*^, Lucy L. Burkitt ^a^, Paramsothy Jeyakumar ^a^, Christopher W. N. Anderson ^a^, Lisanne M. Fermin ^b^ and Daniel J. Donaghy ^a^

*^a^School of Agriculture and Environment, Massey University, Palmerston North, New Zealand.*

*^b^School of Veterinary Science, Massey University, Palmerston North, New Zealand.*

*^c^Department of Agricultural Engineering and Soil Science, Faculty of Agriculture, Rajarata University of Sri Lanka, Sri Lanka.*

*Correspondence:

Upulika Jayaneththi: [H.Jayaneththi@massey.ac.nz](mailto:%20H.Jayaneththi@massey.ac.nz) (ORCID: 0000-0002-9054-6757)

Nicholas W. Sneddon: [N.W.Sneddon@massey.ac.nz](mailto:N.W.Sneddon@massey.ac.nz) (ORCID: 0000-0001-9704-1287)

Supplementary Information: Additional File 1

Table S1: Temporal soil–gut contributions of shared bacterial taxa in grazing dairy cattle across pasture management treatments.

| **Species** | **Treatment** | **Month** | **Soil_reads** | **Gut_reads** | **Total** | **Soil_contrib** | **Gut_contrib** | **Dominance** |
| --- | --- | --- | --- | --- | --- | --- | --- | --- |
| *Akkermansia muciniphila* | Std-Con | August | 781 | 1338 | 2119 | 36.85700802 | 63.14299 | Gut-dominant |
| *Bacteroides uniformis* | Std-Con | August | 921 | 14525 | 15446 | 5.962708792 | 94.03729 | Gut-dominant |
| *Bdellovibrio bacteriovorus* | Std-Con | August | 472 | 23 | 495 | 95.35353535 | 4.646465 | Soil-dominant |
| *Clostridium neonatale* | Std-Con | August | 751 | 811 | 1562 | 48.0793854 | 51.92061 | Gut-dominant |
| *Clostridium perfringens* | Std-Con | August | 560 | 2175 | 2735 | 20.47531993 | 79.52468 | Gut-dominant |
| *Desulfosporosinus meridiei* | Std-Con | August | 387 | 288 | 675 | 57.33333333 | 42.66667 | Soil-dominant |
| *Fibrobacter succinogenes* | Std-Con | August | 27 | 501 | 528 | 5.113636364 | 94.88636 | Gut-dominant |
| *Pseudomonas stutzeri* | Std-Con | August | 1374 | 2113 | 3487 | 39.40349871 | 60.5965 | Gut-dominant |
| *Ruminococcus flavefaciens* | Std-Con | August | 598 | 4858 | 5456 | 10.96041056 | 89.03959 | Gut-dominant |
| *Akkermansia muciniphila* | Div-Reg | August | 2462 | 1315 | 3777 | 65.18400847 | 34.81599 | Soil-dominant |
| *Clostridium neonatale* | Div-Reg | August | 312 | 417 | 729 | 42.79835391 | 57.20165 | Gut-dominant |
| *Clostridium perfringens* | Div-Reg | August | 686 | 3616 | 4302 | 15.94607159 | 84.05393 | Gut-dominant |
| *Fibrobacter succinogenes* | Div-Reg | August | 17 | 1101 | 1118 | 1.520572451 | 98.47943 | Gut-dominant |
| *Prevotella copri* | Div-Reg | August | 372 | 4541 | 4913 | 7.571748423 | 92.42825 | Gut-dominant |
| *Sphingobacterium multivorum* | Div-Reg | August | 332 | 42 | 374 | 88.77005348 | 11.22995 | Soil-dominant |
| *Akkermansia muciniphila* | Div-Con | August | 718 | 2440 | 3158 | 22.7359088 | 77.26409 | Gut-dominant |
| *Bacteroides uniformis* | Div-Con | August | 822 | 11893 | 12715 | 6.464805348 | 93.53519 | Gut-dominant |
| *Bdellovibrio bacteriovorus* | Div-Con | August | 347 | 21 | 368 | 94.29347826 | 5.706522 | Soil-dominant |
| *Clostridium perfringens* | Div-Con | August | 545 | 2007 | 2552 | 21.35579937 | 78.6442 | Gut-dominant |
| *Desulfosporosinus meridiei* | Div-Con | August | 659 | 497 | 1156 | 57.00692042 | 42.99308 | Soil-dominant |
| *Faecalibacterium prausnitzii* | Div-Con | August | 763 | 19771 | 20534 | 3.715788448 | 96.28421 | Gut-dominant |
| *Fibrobacter succinogenes* | Div-Con | August | 28 | 851 | 879 | 3.185437998 | 96.81456 | Gut-dominant |

Table S1 (continued). Temporal soil–gut contributions of shared bacterial taxa in grazing dairy cattle across pasture management treatments.

| **Species** | **Treatment** | **Month** | **Soil_reads** | **Gut_reads** | **Total** | **Soil_contrib** | **Gut_contrib** | **Dominance** |
| --- | --- | --- | --- | --- | --- | --- | --- | --- |
| *Prevotella copri* | Div-Con | August | 956 | 3164 | 4120 | 23.2038835 | 76.79612 | Gut-dominant |
| *Sphingopyxis alaskensis* | Div-Con | August | 545 | 909 | 1454 | 37.48280605 | 62.51719 | Gut-dominant |
| *Variovorax paradoxus* | Div-Con | August | 1438 | 1412 | 2850 | 50.45614035 | 49.54386 | Soil-dominant |
| *Akkermansia muciniphila* | Std-Con | October | 1289 | 916 | 2205 | 58.45804989 | 41.54195 | Soil-dominant |
| *Clostridium neonatale* | Std-Con | October | 2816 | 488 | 3304 | 85.23002421 | 14.76998 | Soil-dominant |
| *Clostridium perfringens* | Std-Con | October | 1704 | 3132 | 4836 | 35.23573201 | 64.76427 | Gut-dominant |
| *Fibrobacter succinogenes* | Std-Con | October | 10 | 659 | 669 | 1.494768311 | 98.50523 | Gut-dominant |
| *Prevotella copri* | Std-Con | October | 1317 | 3056 | 4373 | 30.11662474 | 69.88338 | Gut-dominant |
| *Ruminococcus flavefaciens* | Std-Con | October | 549 | 5576 | 6125 | 8.963265306 | 91.03673 | Gut-dominant |
| *Akkermansia muciniphila* | Div-Reg | October | 2377 | 921 | 3298 | 72.07398423 | 27.92602 | Soil-dominant |
| *Clostridium neonatale* | Div-Reg | October | 472 | 2176 | 2648 | 17.82477341 | 82.17523 | Gut-dominant |
| *Desulfosporosinus meridiei* | Div-Reg | October | 254 | 374 | 628 | 40.44585987 | 59.55414 | Gut-dominant |
| *Fibrobacter succinogenes* | Div-Reg | October | 10 | 1444 | 1454 | 0.687757909 | 99.31224 | Gut-dominant |
| *Akkermansia muciniphila* | Div-Con | October | 1101 | 3614 | 4715 | 23.35100742 | 76.64899 | Gut-dominant |
| *Clostridium neonatale* | Div-Con | October | 654 | 834 | 1488 | 43.9516129 | 56.04839 | Gut-dominant |
| *Clostridium perfringens* | Div-Con | October | 854 | 2393 | 3247 | 26.30120111 | 73.6988 | Gut-dominant |
| *Fibrobacter succinogenes* | Div-Con | October | 21 | 812 | 833 | 2.521008403 | 97.47899 | Gut-dominant |
| *Prevotella copri* | Div-Con | October | 630 | 4211 | 4841 | 13.01384012 | 86.98616 | Gut-dominant |
| *Spirochaeta aurantia* | Div-Con | October | 1439 | 65 | 1504 | 95.67819149 | 4.321809 | Soil-dominant |
| *Akkermansia muciniphila* | Std-Con | November | 873 | 1357 | 2230 | 39.14798206 | 60.85202 | Gut-dominant |
| *Clostridium neonatale* | Std-Con | November | 656 | 1224 | 1880 | 34.89361702 | 65.10638 | Gut-dominant |
| *Fibrobacter succinogenes* | Std-Con | November | 18 | 268 | 286 | 6.293706294 | 93.70629 | Gut-dominant |

Table S1 (continued). Temporal soil–gut contributions of shared bacterial taxa in grazing dairy cattle across pasture management treatments.

| **Species** | **Treatment** | **Month** | **Soil_reads** | **Gut_reads** | **Total** | **Soil_contrib %** | **Gut_contrib %** | **Dominance** |
| --- | --- | --- | --- | --- | --- | --- | --- | --- |
| *Akkermansia muciniphila* | Div-Reg | November | 876 | 2210 | 3086 | 28.38626053 | 71.61374 | Gut-dominant |
| *Clostridium hiranonis* | Div-Reg | November | 486 | 839 | 1325 | 36.67924528 | 63.32075 | Gut-dominant |
| *Clostridium neonatale* | Div-Reg | November | 1371 | 1294 | 2665 | 51.44465291 | 48.55535 | Soil-dominant |
| *Clostridium perfringens* | Div-Reg | November | 738 | 1333 | 2071 | 35.63495896 | 64.36504 | Gut-dominant |
| *Collinsella aerofaciens* | Div-Reg | November | 2680 | 5764 | 8444 | 31.73851255 | 68.26149 | Gut-dominant |
| *Desulfosporosinus meridiei* | Div-Reg | November | 1040 | 455 | 1495 | 69.56521739 | 30.43478 | Soil-dominant |
| *Fibrobacter succinogenes* | Div-Reg | November | 44 | 890 | 934 | 4.710920771 | 95.28908 | Gut-dominant |
| *Prevotella copri* | Div-Reg | November | 636 | 5786 | 6422 | 9.903456867 | 90.09654 | Gut-dominant |
| *Akkermansia muciniphila* | Div-Con | November | 1250 | 2368 | 3618 | 34.54947485 | 65.45053 | Gut-dominant |
| *Clostridium neonatale* | Div-Con | November | 1088 | 1009 | 2097 | 51.8836433 | 48.11636 | Soil-dominant |
| *Clostridium perfringens* | Div-Con | November | 1337 | 1365 | 2702 | 49.48186528 | 50.51813 | Gut-dominant |
| *Fibrobacter succinogenes* | Div-Con | November | 14 | 919 | 933 | 1.500535906 | 98.49946 | Gut-dominant |
| *Prevotella copri* | Div-Con | November | 714 | 5322 | 6036 | 11.82902584 | 88.17097 | Gut-dominant |
| *Akkermansia muciniphila* | Std-Con | December | 1200 | 916 | 2116 | 56.71077505 | 43.28922 | Soil-dominant |
| *Clostridium neonatale* | Std-Con | December | 863 | 488 | 1351 | 63.87860844 | 36.12139 | Soil-dominant |
| *Clostridium perfringens* | Std-Con | December | 1388 | 3132 | 4520 | 30.7079646 | 69.29204 | Gut-dominant |
| *Fibrobacter succinogenes* | Std-Con | December | 44 | 659 | 703 | 6.258890469 | 93.74111 | Gut-dominant |
| *Prevotella copri* | Std-Con | December | 444 | 3056 | 3500 | 12.68571429 | 87.31429 | Gut-dominant |
| *Akkermansia muciniphila* | Div-Reg | December | 804 | 921 | 1725 | 46.60869565 | 53.3913 | Gut-dominant |
| *Clostridium neonatale* | Div-Reg | December | 760 | 2176 | 2936 | 25.88555858 | 74.11444 | Gut-dominant |
| *Clostridium perfringens* | Div-Reg | December | 1327 | 2435 | 3762 | 35.27379054 | 64.72621 | Gut-dominant |
| *Desulfosporosinus meridiei* | Div-Reg | December | 299 | 374 | 673 | 44.42793462 | 55.57207 | Gut-dominant |

Table S1 (continued). Temporal soil–gut contributions of shared bacterial taxa in grazing dairy cattle across pasture management treatments.

| **Species** | **Treatment** | **Month** | **Soil_reads** | **Gut_reads** | **Total** | **Soil_contrib** | **Gut_contrib** | **Dominance** |
| --- | --- | --- | --- | --- | --- | --- | --- | --- |
| *Fibrobacter succinogenes* | Div-Reg | December | 21 | 1444 | 1465 | 1.433447099 | 98.56655 | Gut-dominant |
| *Prevotella copri* | Div-Reg | December | 792 | 3328 | 4120 | 19.22330097 | 80.7767 | Gut-dominant |
| *Akkermansia muciniphila* | Div-Con | December | 750 | 3614 | 4364 | 17.18606783 | 82.81393 | Gut-dominant |
| *Clostridium neonatale* | Div-Con | December | 1189 | 834 | 2023 | 58.77409787 | 41.2259 | Soil-dominant |
| *Clostridium perfringens* | Div-Con | December | 1243 | 2393 | 3636 | 34.18591859 | 65.81408 | Gut-dominant |
| *Fibrobacter succinogenes* | Div-Con | December | 50 | 812 | 862 | 5.800464037 | 94.19954 | Gut-dominant |
| *Prevotella copri* | Div-Con | December | 1139 | 4211 | 5350 | 21.28971963 | 78.71028 | Gut-dominant |
| *Prevotella stercorea* | Div-Con | December | 222 | 613 | 835 | 26.58682635 | 73.41317 | Gut-dominant |
| *Spirochaeta aurantia* | Div-Con | December | 511 | 65 | 576 | 88.71527778 | 11.28472 | Soil-dominant |
| *Akkermansia muciniphila* | Std-Con | February | 959 | 2185 | 3144 | 30.50254453 | 69.49746 | Gut-dominant |
| *Clostridium neonatale* | Std-Con | February | 2798 | 1467 | 4265 | 65.60375147 | 34.39625 | Soil-dominant |
| *Fibrobacter succinogenes* | Std-Con | February | 83 | 1239 | 1322 | 6.278366112 | 93.72163 | Gut-dominant |
| *Prevotella copri* | Std-Con | February | 790 | 4456 | 5246 | 15.05909264 | 84.94091 | Gut-dominant |
| *Clostridium neonatale* | Div-Reg | February | 914 | 1228 | 2142 | 42.67040149 | 57.3296 | Gut-dominant |
| *Desulfosporosinus meridiei* | Div-Reg | February | 660 | 557 | 1217 | 54.23171734 | 45.76828 | Soil-dominant |
| *Fibrobacter succinogenes* | Div-Reg | February | 39 | 159 | 198 | 19.6969697 | 80.30303 | Gut-dominant |
| *Flavobacterium succinicans* | Div-Reg | February | 362 | 98 | 460 | 78.69565217 | 21.30435 | Soil-dominant |
| *Lysinibacillus boronitolerans* | Div-Reg | February | 1037 | 1126 | 2163 | 47.94267221 | 52.05733 | Gut-dominant |
| *Prevotella copri* | Div-Reg | February | 502 | 5353 | 5855 | 8.573868488 | 91.42613 | Gut-dominant |
| *Ruminococcus flavefaciens* | Div-Reg | February | 576 | 10861 | 11437 | 5.036285739 | 94.96371 | Gut-dominant |
| *Akkermansia muciniphila* | Div-Con | February | 622 | 1952 | 2574 | 24.16472416 | 75.83528 | Gut-dominant |
| *Bacteroides fragilis* | Div-Con | February | 1364 | 2455 | 3819 | 35.71615606 | 64.28384 | Gut-dominant |

Table S1 (continued). Temporal soil–gut contributions of shared bacterial taxa in grazing dairy cattle across pasture management treatments.

| **Species** | **Treatment** | **Month** | **Soil_reads** | **Gut_reads** | **Total** | **Soil_contrib** | **Gut_contrib** | **Dominance** |
| --- | --- | --- | --- | --- | --- | --- | --- | --- |
| *Clostridium perfringens* | Div-Con | February | 1119 | 1395 | 2514 | 44.51073986 | 55.48926 | Gut-dominant |
| *Faecalibacterium prausnitzii* | Div-Con | February | 1064 | 20894 | 21958 | 4.845614355 | 95.15439 | Gut-dominant |
| *Fibrobacter succinogenes* | Div-Con | February | 139 | 491 | 630 | 22.06349206 | 77.93651 | Gut-dominant |
| *Parabacteroides distasonis* | Div-Con | February | 371 | 1178 | 1549 | 23.95093609 | 76.04906 | Gut-dominant |
| *Prevotella copri* | Div-Con | February | 1762 | 2830 | 4592 | 38.37108014 | 61.62892 | Gut-dominant |
| *Pseudomonas stutzeri* | Div-Con | February | 2045 | 1422 | 3467 | 58.98471301 | 41.01529 | Soil-dominant |
| *Ruminococcus flavefaciens* | Div-Con | February | 738 | 5990 | 6728 | 10.96908442 | 89.03092 | Gut-dominant |
| *Akkermansia muciniphila* | Std-Con | April | 519 | 3256 | 3775 | 13.74834437 | 86.25166 | Gut-dominant |
| *Bacteroides uniformis* | Std-Con | April | 1241 | 10764 | 12005 | 10.33735943 | 89.66264 | Gut-dominant |
| *Clostridium neonatale* | Std-Con | April | 1041 | 668 | 1709 | 60.91281451 | 39.08719 | Soil-dominant |
| *Clostridium perfringens* | Std-Con | April | 764 | 407 | 1171 | 65.24338173 | 34.75662 | Soil-dominant |
| *Fibrobacter succinogenes* | Std-Con | April | 45 | 8 | 53 | 84.90566038 | 15.09434 | Soil-dominant |
| *Prevotella copri* | Std-Con | April | 614 | 2698 | 3312 | 18.53864734 | 81.46135 | Gut-dominant |
| *Bacteroides uniformis* | Div-Reg | April | 1090 | 5494 | 6584 | 16.55528554 | 83.44471 | Gut-dominant |
| *Clostridium perfringens* | Div-Reg | April | 1361 | 658 | 2019 | 67.40960872 | 32.59039 | Soil-dominant |
| *Fibrobacter succinogenes* | Div-Reg | April | 38 | 471 | 509 | 7.465618861 | 92.53438 | Gut-dominant |
| *Prevotella copri* | Div-Reg | April | 559 | 2520 | 3079 | 18.15524521 | 81.84475 | Gut-dominant |
| *Akkermansia muciniphila* | Div-Con | April | 883 | 1952 | 2835 | 31.14638448 | 68.85362 | Gut-dominant |
| *Clostridium perfringens* | Div-Con | April | 3944 | 1395 | 5339 | 73.87151152 | 26.12849 | Soil-dominant |
| *Faecalibacterium prausnitzii* | Div-Con | April | 1459 | 20894 | 22353 | 6.527088087 | 93.47291 | Gut-dominant |
| *Fibrobacter succinogenes* | Div-Con | April | 40 | 491 | 531 | 7.532956685 | 92.46704 | Gut-dominant |
| *Prevotella copri* | Div-Con | April | 592 | 2830 | 3422 | 17.29982466 | 82.70018 | Gut-dominant |

Std-Con: Standard pastures under contemporary management; Div-Reg: Diverse pastures under regenerative management; Div-Con: Diverse pastures under contemporary management.
