# Supplementary material for: Host Filtering Shapes the Soil–gut Microbiome Linkages in Pastoral Systems
Source: Microb Ecol. 2026 May 21;89(1):146. doi: 10.1007/s00248-026-02791-6 (PMC13369704; doi:10.1007/s00248-026-02791-6)
Supplement: Supplementary file 2 — Supplementary Material 2 (DOCX 35.0 KB) [file 248_2026_2791_MOESM2_ESM.docx]

**Host filtering shapes the soil–gut microbiome linkages in pastoral systems**

Upulika Jayaneththi ^a,c^*, Nicholas W. Sneddon ^a*^, Lucy L. Burkitt ^a^, Paramsothy Jeyakumar ^a^, Christopher W. N. Anderson ^a^, Lisanne M. Fermin ^b^ and Daniel J. Donaghy ^a^

*^a^School of Agriculture and Environment, Massey University, Palmerston North, New Zealand.*

*^b^School of Veterinary Science, Massey University, Palmerston North, New Zealand.*

*^c^Department of Agricultural Engineering and Soil Science, Faculty of Agriculture, Rajarata University of Sri Lanka, Sri Lanka.*

*Correspondence:

Upulika Jayaneththi: [H.Jayaneththi@massey.ac.nz](mailto:%20H.Jayaneththi@massey.ac.nz) (ORCID: 0000-0002-9054-6757)

Nicholas W. Sneddon: [N.W.Sneddon@massey.ac.nz](mailto:N.W.Sneddon@massey.ac.nz) (ORCID: 0000-0001-9704-1287)

Supplementary Information: Additional File 2

Table S2: Temporal soil–gut contributions of shared bacterial taxa in grazing sheep across pasture management treatments.

| **Species** | | | **Treatment** | | **Month** | **Soil_reads** | | **Gut_reads** | | **Total** | | **Soil_contrib%** | | **Gut_contrib%** | | **Dominance** | |
| --- | --- | --- | --- | --- | --- | --- | --- | --- | --- | --- | --- | --- | --- | --- | --- | --- | --- |
| *Clostridium perfringens* | | | Std-Con | | March | 680 | | 1095 | | 1775 | | 38.30986 | | 61.69014 | | Gut-dominant | |
| *Akkermansia muciniphila* | | | Std-Con | | March | 922 | | 2103 | | 3025 | | 30.47934 | | 69.52066 | | Gut-dominant | |
| *Akkermansia muciniphila* | | | Std-Reg | | March | 1490 | | 3648 | | 5138 | | 28.99961 | | 71.00039 | | Gut-dominant | |
| *Clostridium neonatale* | | | Std-Reg | | March | 1071 | | 406 | | 1477 | | 72.51185 | | 27.48815 | | Soil-dominant | |
| *Clostridium perfringens* | | | Std-Reg | | March | 1321 | | 2855 | | 4176 | | 31.63314 | | 68.36686 | | Gut-dominant | |
| *Desulfovibrio mexicanus* | | | Std-Reg | | March | 73 | | 104 | | 177 | | 41.24294 | | 58.75706 | | Gut-dominant | |
| *Akkermansia muciniphila* | | | Div-Con | | March | 462 | | 3919 | | 4381 | | 10.54554 | | 89.45446 | | Gut-dominant | |
| *Anoxybacillus kestanbolensis* | | | Div-Con | | March | 91 | | 187 | | 278 | | 32.73381 | | 67.26619 | | Gut-dominant | |
| *Clostridium perfringens* | | | Div-Con | | March | 1499 | | 11060 | | 12559 | | 11.93566 | | 88.06434 | | Gut-dominant | |
| *Desulfovibrio mexicanus* | | | Div-Con | | March | 20 | | 111 | | 131 | | 15.26718 | | 84.73282 | | Gut-dominant | |
| *Akkermansia muciniphila* | | | Div-Reg | | March | 2020 | | 5153 | | 7173 | | 28.16116 | | 71.83884 | | Gut-dominant | |
| *Clostridium neonatale* | | | Div-Reg | | March | 1307 | | 872 | | 2179 | | 59.98164 | | 40.01836 | | Soil-dominant | |
| *Clostridium perfringens* | | | Div-Reg | | March | 1965 | | 1979 | | 3944 | | 49.82252 | | 50.17748 | | Gut-dominant | |
| *Desulfovibrio mexicanus* | | | Div-Reg | | March | 99 | | 82 | | 181 | | 54.69613 | | 45.30387 | | Soil-dominant | |
| *Propionibacterium acnes* | | | Div-Reg | | March | 678 | | 222 | | 900 | | 75.33333 | | 24.66667 | | Soil-dominant | |
| *Akkermansia muciniphila* | | | Std-Con | | June | 732 | | 2522 | | 3254 | | 22.49539 | | 77.50461 | | Gut-dominant | |
| *Clostridium perfringens* | | | Std-Con | | June | 1578 | | 2110 | | 3688 | | 42.78742 | | 57.21258 | | Gut-dominant | |
| *Desulfovibrio mexicanus* | | | Std-Con | | June | 42 | | 116 | | 158 | | 26.58228 | | 73.41772 | | Gut-dominant | |
| *Fibrobacter succinogenes* | | | Std-Con | | June | 9 | | 3263 | | 3272 | | 0.275061 | | 99.72494 | | Gut-dominant | |
| *Akkermansia muciniphila* | | | Std-Reg | | June | 1834 | | 1727 | | 3561 | | 51.50239 | | 48.49761 | | Soil-dominant | |
| *Clostridium perfringens* | | | Std-Reg | | June | 3260 | | 797 | | 4057 | | 80.35494 | | 19.64506 | | Soil-dominant | |
| *Fibrobacter succinogenes* | | | Std-Reg | | June | 51 | | 5200 | | 5251 | | 0.971244 | | 99.02876 | | Gut-dominant | |
| Table S2 (continued). Temporal soil–gut contributions of shared bacterial taxa in grazing sheep across pasture management treatments. | | | | | | | | | | | | | | | | | |
| **Species** | | **Treatment** | | **Month** | | **Soil_reads** | | **Gut_reads** | | **Total** | | **Soil_contrib%** | | **Gut_contrib%** | | **Dominance** | |
| *Pseudomonas stutzeri* | | Std-Reg | | June | | 1198 | | 883 | | 2081 | | 57.56848 | | 42.43152 | | Soil-dominant | |
| *Prevotella copri* | | Std-Reg | | June | | 1002 | | 6858 | | 7860 | | 12.74809 | | 87.25191 | | Gut-dominant | |
| *Akkermansia muciniphila* | | Div-Con | | June | | 407 | | 4024 | | 4431 | | 9.185285 | | 90.81471 | | Gut-dominant | |
| *Clostridium perfringens* | | Div-Con | | June | | 599 | | 820 | | 1419 | | 42.21283 | | 57.78717 | | Gut-dominant | |
| *Desulfosporosinus meridiei* | | Div-Con | | June | | 136 | | 172 | | 308 | | 44.15584 | | 55.84416 | | Gut-dominant | |
| *Akkermansia muciniphila* | | Div-Reg | | June | | 979 | | 2333 | | 3312 | | 29.55918 | | 70.44082 | | Gut-dominant | |
| *Clostridium perfringens* | | Div-Reg | | June | | 753 | | 7494 | | 8247 | | 9.130593 | | 90.86941 | | Gut-dominant | |
| *Acholeplasma laidlawii* | | Std-Con | | August | | 21 | | 8 | | 29 | | 72.41379 | | 27.58621 | | Soil-dominant | |
| *Bacteroides uniformis* | | Std-Con | | August | | 1286 | | 2543 | | 3829 | | 33.58579 | | 66.41421 | | Gut-dominant | |
| *Clostridium perfringens* | | Std-Con | | August | | 878 | | 10812 | | 11690 | | 7.510693 | | 92.48931 | | Gut-dominant | |
| *Akkermansia muciniphila* | | Std-Con | | August | | 975 | | 1017 | | 1992 | | 48.94578 | | 51.05422 | | Gut-dominant | |
| *Faecalibacterium prausnitzii* | | Std-Con | | August | | 1747 | | 19305 | | 21052 | | 8.298499 | | 91.7015 | | Gut-dominant | |
| *Fibrobacter succinogenes* | | Std-Con | | August | | 65 | | 5547 | | 5612 | | 1.158232 | | 98.84177 | | Gut-dominant | |
| *Akkermansia muciniphila* | | Std-Reg | | August | | 1717 | | 831 | | 2548 | | 67.38619 | | 32.61381 | | Soil-dominant | |
| *Clostridium neonatale* | | Std-Reg | | August | | 981 | | 614 | | 1595 | | 61.5047 | | 38.4953 | | Soil-dominant | |
| *Clostridium perfringens* | | Std-Reg | | August | | 2562 | | 5197 | | 7759 | | 33.01972 | | 66.98028 | | Gut-dominant | |
| *Desulfovibrio mexicanus* | | Std-Reg | | August | | 14 | | 94 | | 108 | | 12.96296 | | 87.03704 | | Gut-dominant | |
| *Fibrobacter succinogenes* | | Std-Reg | | August | | 48 | | 5917 | | 5965 | | 0.804694 | | 99.19531 | | Gut-dominant | |
| *Parabacteroides distasonis* | | Std-Reg | | August | | 204 | | 7905 | | 8109 | | 2.515723 | | 97.48428 | | Gut-dominant | |
| *Akkermansia muciniphila* | | Div-Con | | August | | 986 | | 1781 | | 2767 | | 35.63426 | | 64.36574 | | Gut-dominant | |
| *Clostridium neonatale* | | Div-Con | | August | | 734 | | 820 | | 1554 | | 47.23295 | | 52.76705 | | Gut-dominant | |
| *Clostridium perfringens* | | Div-Con | | August | | 1361 | | 5438 | | 6799 | | 20.01765 | | 79.98235 | | Gut-dominant | |
| Table S2 (continued). Temporal soil–gut contributions of shared bacterial taxa in grazing sheep across pasture management treatments. | | | | | | | | | | | | | | | | | |
| **Species** | **Treatment** | | | | **Month** | | **Soil_reads** | | **Gut_reads** | | **Total** | | **Soil_contrib%** | | **Gut_contrib%** | | **Dominance** |
| *Prevotella copri* | Div-Con | | | | August | | 849 | | 3519 | | 4368 | | 19.43681 | | 80.56319 | | Gut-dominant |
| *Sulfuricurvum kujiense* | Div-Con | | | | August | | 13 | | 21 | | 34 | | 38.23529 | | 61.76471 | | Gut-dominant |
| *Fibrobacter succinogenes* | Div-Con | | | | August | | 17 | | 2795 | | 2812 | | 0.604552 | | 99.39545 | | Gut-dominant |
| *Eubacterium dolichum* | Div-Reg | | | | August | | 22 | | 204 | | 226 | | 9.734513 | | 90.26549 | | Gut-dominant |
| *Akkermansia muciniphila* | Div-Reg | | | | August | | 2686 | | 2333 | | 5019 | | 53.51664 | | 46.48336 | | Soil-dominant |
| *Bacteroides fragilis* | Div-Reg | | | | August | | 405 | | 7577 | | 7982 | | 5.073916 | | 94.92608 | | Gut-dominant |
| *Clostridium neonatale* | Div-Reg | | | | August | | 940 | | 306 | | 1246 | | 75.44141 | | 24.55859 | | Soil-dominant |
| *Clostridium perfringens* | Div-Reg | | | | August | | 1013 | | 16158 | | 17171 | | 5.899482 | | 94.10052 | | Gut-dominant |
| *Akkermansia muciniphila* | Std-Con | | | | October | | 1369 | | 1367 | | 2736 | | 50.03655 | | 49.96345 | | Soil-dominant |
| *Clostridium neonatale* | Std-Con | | | | October | | 338 | | 327 | | 665 | | 50.82707 | | 49.17293 | | Soil-dominant |
| *Clostridium perfringens* | Std-Con | | | | October | | 721 | | 12156 | | 12877 | | 5.59913 | | 94.40087 | | Gut-dominant |
| *Desulfovibrio mexicanus* | Std-Con | | | | October | | 41 | | 132 | | 173 | | 23.69942 | | 76.30058 | | Gut-dominant |
| *Fibrobacter succinogenes* | Std-Con | | | | October | | 6 | | 3917 | | 3923 | | 0.152944 | | 99.84706 | | Gut-dominant |
| *Akkermansia muciniphila* | Std-Reg | | | | October | | 1991 | | 3443 | | 5434 | | 36.63968 | | 63.36032 | | Gut-dominant |
| *Clostridium perfringens* | Std-Reg | | | | October | | 824 | | 4107 | | 4931 | | 16.71061 | | 83.28939 | | Gut-dominant |
| *Desulfovibrio mexicanus* | Std-Reg | | | | October | | 38 | | 237 | | 275 | | 13.81818 | | 86.18182 | | Gut-dominant |
| *Fibrobacter succinogenes* | Std-Reg | | | | October | | 14 | | 631 | | 645 | | 2.170543 | | 97.82946 | | Gut-dominant |
| *Prevotella copri* | Std-Reg | | | | October | | 399 | | 3978 | | 4377 | | 9.115833 | | 90.88417 | | Gut-dominant |
| *Akkermansia muciniphila* | Div-Con | | | | October | | 1670 | | 3040 | | 4710 | | 35.45648 | | 64.54352 | | Gut-dominant |
| *Anoxybacillus kestanbolensis* | Div-Con | | | | October | | 86 | | 151 | | 237 | | 36.28692 | | 63.71308 | | Gut-dominant |
| *Clostridium neonatale* | Div-Con | | | | October | | 461 | | 502 | | 963 | | 47.87124 | | 52.12876 | | Gut-dominant |
| *Clostridium perfringens* | Div-Con | | | | October | | 750 | | 8945 | | 9695 | | 7.735946 | | 92.26405 | | Gut-dominant |
| *Desulfovibrio mexicanus* | Div-Con | | | | October | | 142 | | 118 | | 260 | | 54.61538 | | 45.38462 | | Soil-dominant |
| *Fibrobacter succinogenes* | Div-Con | | | | October | | 11 | | 2744 | | 2755 | | 0.399274 | | 99.60073 | | Gut-dominant |
| *Akkermansia muciniphila* | Div-Reg | | | | October | | 1455 | | 3117 | | 4572 | | 31.82415 | | 68.17585 | | Gut-dominant |
| *Clostridium perfringens* | Div-Reg | | | | October | | 1442 | | 2163 | | 3605 | | 40 | | 60 | | Gut-dominant |
| *Desulfovibrio mexicanus* | Div-Reg | | | | October | | 40 | | 193 | | 233 | | 17.16738 | | 82.83262 | | Gut-dominant |
| *Fibrobacter succinogenes* | Div-Reg | | | | October | | 23 | | 527 | | 550 | | 4.181818 | | 95.81818 | | Gut-dominant |
| *Prevotella copri* | Div-Reg | | | | October | | 647 | | 6412 | | 7059 | | 9.165604 | | 90.8344 | | Gut-dominant |

Table S2 (continued). Temporal soil–gut contributions of shared bacterial taxa in grazing sheep across pasture management treatments.

| **Species** | **Treatment** | **Month** | **Soil_reads** | **Gut_reads** | **Total** | **Soil_contrib**  **%** | **Gut_contrib**  **%** | **Dominance** |
| --- | --- | --- | --- | --- | --- | --- | --- | --- |
| *Ruminococcus flavefaciens* | Div-Reg | October | 337 | 15364 | 15701 | 2.14636 | 97.85364 | Gut-dominant |
| *Akkermansia muciniphila* | Std-Con | November | 739 | 7394 | 8133 | 9.086438 | 90.91356 | Gut-dominant |
| *Clostridium neonatale* | Std-Con | November | 287 | 488 | 775 | 37.03226 | 62.96774 | Gut-dominant |
| *Clostridium perfringens* | Std-Con | November | 1013 | 16127 | 17140 | 5.910152 | 94.08985 | Gut-dominant |
| *Desulfovibrio mexicanus* | Std-Con | November | 130 | 175 | 305 | 42.62295 | 57.37705 | Gut-dominant |
| *Fibrobacter succinogenes* | Std-Con | November | 9 | 58 | 67 | 13.43284 | 86.56716 | Gut-dominant |
| *Lysinibacillus boronitolerans* | Std-Con | November | 664 | 978 | 1642 | 40.43849 | 59.56151 | Gut-dominant |
| *Akkermansia muciniphila* | Std-Reg | November | 1007 | 2687 | 3694 | 27.26042 | 72.73958 | Gut-dominant |
| *Clostridium neonatale* | Std-Reg | November | 723 | 285 | 1008 | 71.72619 | 28.27381 | Soil-dominant |
| *Clostridium perfringens* | Std-Reg | November | 1184 | 11478 | 12662 | 9.350813 | 90.64919 | Gut-dominant |
| *Fibrobacter succinogenes* | Std-Reg | November | 13 | 4450 | 4463 | 0.291284 | 99.70872 | Gut-dominant |
| *Akkermansia muciniphila* | Div-Con | November | 970 | 2522 | 3492 | 27.77778 | 72.22222 | Gut-dominant |
| *Bacteroides uniformis* | Div-Con | November | 1144 | 2455 | 3599 | 31.78661 | 68.21339 | Gut-dominant |
| *Fibrobacter succinogenes* | Div-Con | November | 28 | 1619 | 1647 | 1.700061 | 98.29994 | Gut-dominant |
| *Prevotella copri* | Div-Con | November | 908 | 3993 | 4901 | 18.52683 | 81.47317 | Gut-dominant |
| *Akkermansia muciniphila* | Div-Reg | November | 676 | 4336 | 5012 | 13.48763 | 86.51237 | Gut-dominant |
| *Variovorax paradoxus* | Div-Reg | November | 1519 | 672 | 2191 | 69.32907 | 30.67093 | Soil-dominant |

Std-Con: Standard pastures under contemporary management; Std-Reg: Standard Pastures under Regenerative management; Div-Con: Diverse pastures under contemporary management; Div-Reg: Diverse pastures under regenerative management.
